# Supplementary material for: NASH limits anti-tumour surveillance in immunotherapy-treated HCC
Source: Nature. 2021 Mar 24;592(7854):450–6. doi: 10.1038/s41586-021-03362-0 (PMC8046670; doi:10.1038/s41586-021-03362-0)
Supplement: Supplementary file 1 — This file contains Supplementary Tables 1-15. [file 41586_2021_3362_MOESM1_ESM.pdf]

nature portfolio

https://doi.org/10.1038/s41586-021-03362-0

Supplementary information

NASH limits anti-tumour surveillance in immunotherapy-treated HCC

In the format provided by the authors and unedited

Supplementary Table 1: Demographics patients included for flow cytometry analysis of human patients in Extended Data 9a-e

	Control	NAFLD/NASH
Age (years), mean±SD	46.5±14.1	55.3±10.2
Sex		
Male	3 (37.5 %)	10 (62.5 %)
Female	5 (62.5 %)	6 (37.5%)
BMI	21.0±2.8	30.5±5.3
Obesity (BMI>30)	0 (0.0 %)	8 (50.0 %)
macrovesicular Steatosis in % (cut-off 5%)	<5.0±0.0	48.4±25.8
NAS (0-8)	0.0±0.0	3.3±1.4
Fibrosis (0-4)	0.1±0.3	1.6±1.4

Supplementary Table 2: Demographics patients included for flow cytometry analysis of human patients in Figure 3a,b and Extendend Data 9f-g

	Control	NAFLD/NASH
Age (years), mean±SD	45.2±6.5	52.6±14.7
Sex		
Male	1 (16.67 %)	6 (54.5%)
Female	5 (83.33 %)	5 (45.5 %)
BMI	41.0±9.8	40.5±10.2
Obesity (BMI≥30)	5 (83.33 %)	8 (72.7 %)
macrovesicular Steatosis in % (cut-off 5%)	<5.0±1.9	22.7±17.5
NAS (0-8)	1.5±0.5	3.8±0.9
Fibrosis (0-4)	1.2±0.4	1.8±0.4

Supplementary Table 3: Demographics patients included for immunohistochemistry for progressive NAFLD/NASH

Sex (% female)	35
Age (average, years)	55.5
BMI (average)	31.1
T2DM (%)	67.7
Disease stage (n-value)	
NAFL	9
NASH F0/1	7
NASH F2	12
NASH F3	21
NASH F4	16
NAS score (0-8)	4.4

Supplementary Table 4: Quantification of PD-1$^+$ lymphocytes and percentage of CD8$^+$ lymphocytes/total lymphocytes count.

	**PD-1 positive cases (cut-off 1%)**	**PD-1 positive cases/cm2, median (range)**	**Ratio CD8 positive /total lymphocytes (%)**
Healthy (n=4)	0 (0%)	0 (0)	2.00%
NASH (n=26)	5 (19.2%)	0.1 (0 - 4.18)	26.10%
Non-tumor adjacent NASH-HCC (n=16)	3 (18.7%)	0.43 (0 - 2.4)	14.50%
Non-tumor adjacent HCC other etiologies (n=29)	0 (0%)	0 (0 - 0.06)	37.60%

Supplementary Table 5: Quantification of immune infiltration in different types of liver samples. p values: NASH vs. non-tumor adjacent NASH-HCC p= 0.0012; non-tumor adjacent NASH-HCC vs. non-tumor adjacent non-NASH-HCC p= 0.021.

	**Healthy (n=4)**	**NASH (n=26)**	**Peri-tumoral NASH-HCC (n=16)**	**Peri-tumoral HCC other etiologies (n=29)**
Absent	4 (100%)	0 (0%)	0 (0%)	0 (0%)
Mild	0 (0%)	9 (34.6%)	14 (87.5%)	14 (48.3%)
Moderate	0 (0%)	17 (65.4%)	2 (12.5%)	15 (51.7%)
Marked	0 (0%)	0 (0%)	0 (0%)	0 (0%)

Supplementary Table 6: Distribution of cirrhosis in the study cohort.

	**NASH (n=26)**	**Peri-tumoral NASH-HCC (n=16)**	**Peri-tumoral Non-NASH-HCC (n=29)**
F4 (cirrhosis)	25 (96.2%)	13 (81.3%)	22 (75.9%)
non- F4 (F1-F2-F3)	1 (3.8%)	3 (18.7%)	7 (24.1%)

Supplementary Table 7: Pooled baseline characteristics of the patients included in the meta-analysis (total n= 1657).

Trial:	CheckMate 459		KEYNOTE-240		IMbrave150		Total	
Treatment	ICI<br>Nivo	Control<br>Sora	ICI<br>Pembro	Control<br>Placebo	ICI<br>A+B	Control<br>Sora	ICI	Control
**Number of patients**	371	372	278	135	336	165	985	672
**Age, median**	65	65	67	65	64	66	-	-
**Male, n (%)**	314 (85)	317 (85)	226 (81)	112 (83)	277 (82)	137 (83)	817 (83)	566 (84)
**Region**								
Asia, n (%)	147 (40)	148 (40)	67 (24)	31 (23)	133 (40)	68 (41)	347 (35)	247 (37)
Rest, n (%)	224 (60)	224 (60)	211 (76)	104 (77)	203 (60)	97 (59)	638 (65)	425 (63)
**ECOG PS-1, n (%)**	99 (27)	111 (30)	116 (42)	64 (47)	127 (38)	62 (38)	342 (35)	237 (35)
**BCLC C, n (%)**	303 (82)	291 (78)	222 (80)	106 (79)	276 (82)	133 (81)	801 (81)	530 (79)
**Etiology***								
Non-viral, n (%)	168 (45)	168 (45)	163 (59)	85 (63)	100 (30)	53 (32)	431 (44)	306 (46)
HBV, n (%)	116 (31)	117 (31)	72 (26)	29 (22)	164 (49)	76 (46)	352 (36)	222 (33)
HCV, n (%)	87 (23)	86 (23)	43 (16)	21 (16)	72 (21)	36 (22)	202 (21)	143 (21)

Abbreviations: Nivo: Nivolumab, Sora: Sorafenib, Pembro: Pembrolizumab, A + B: atezolizumab + bevacizumab, ICI: immune checkpoint inhibitor, n: number of patients.

* One patient in the CheckMate 459 control arm had an unknown disease etiology.

Supplementary Table 8: Baseline characteristics of 130 HCC patients treated with anti-PD-(L)1-based immunotherapy

	**NAFLD, n=13**	**Other etiology, n=117**	**p-value**
**Age (years), mean±SD**	72.5±8.5	67.4±8.6	0.046
**Sex**			0.692
Male	12 (92%)	97 (83%)	
Female	1 (8%)	20 (17%)	
**Body mass index (kg/m^2), median IIQR)**	23.3 (22.8-26.3)	24.2 (22.3-28.4)	0.981
**Child-Pugh stage**			0.229
A	6 (46%)	76 (65%)	
B	7 (54%)	41 (35%)	
**Performance status**			0.723
0	5 (39%)	51 (44%)	
≥1	8 (62%)	66 (56%)	
**Type of immunotherapy**			0.037
PD-(L)1 Monotherapy	9 (69%)	86 (74%)	
Atezolizumab + bevacizumab	3 (23%)	6 (5%)	
Combination therapies	1 (8%)	25 (21%)	

**Immunotherapy as systemic**			0.092
First-line	6 (46%)	27 (23%)	
Second-/third-line	7 (54%)	90 (77%)	
**Macrovascular invasion**	3 (23%)	57 (49%)	0.079
**Extrahepatic metastasis**	10 (77%)	78 (67%)	0.547
**Alpha-fetoprotein (ng/ml)**			0.234
≤200	8 (62%)	49 (42%)	
>200	5 (39%)	62 (53%)	
**AST (U/l) median (IQR)**	56 (40-90)	63 (39-109)	1.000
**ALT (U/l) median±IQR**	35 (24-54)	38 (27-58)	0.807
Abbreviations: AST, aspartate aminotransferase; ALT, alanine aminotransferase; BCLC, Barcelona-Clinic Liver Cancer; PS, Performance Status; HCC, hepatocellular carcinoma; NAFLD, non-alcoholic fatty liver disease			

Supplementary Table 9: Multivariable analysis of prognostic factors in HCC patients treated with anti-PD-(L)1-based immunotherapy

		Overall survival		
		**HR**	**95% CI**	**p-value (Cox regression)**
**Etiology**	**Other etiologies**	1		0.017
	**NAFLD**	2.6	1.2-5.6	
**Performance Status**	**0**<br>**≥1**	1<br>1.7	 1.0-2.8	0.049
**Macrovascular invasion**	**Absent**<br>**Present t**	1<br>1.8	 1.1-3.0	0.016
**Extrahepatic metastases**	**Absent**<br>**Present**	1<br>0.7	 0.4-1.1	0.121
**Alpha-fetoprotein (ng/ml)**	≤200<br>>200	1<br>1.8	 1.1-2.9	0.019
**Child-Pugh class**	**A**<br>**B**	1<br>1.6	 1.0-2.6	0.075
**Confounder for survival (months)**	**Age**	**<65 years**<br>**>65 years**	16.2 (9.1-23.4)<br>10.1 (7.7-12.5)	0.193
	**Gender**	**Male**<br>**Female**	11.1 (7.5-14.7)<br>8.9 (5.5-12.4)	0.455

Supplementary Table 10: Baseline characteristics of 118 HCC patients treated with anti-PD-(L)1-based immunotherapy

	**NAFLD, n=11**	**Other etiology, n=107**	**p-value**
**Age (years), mean±SD**	67.3±7.5	61.9±10.1	0.046
**Sex**			0.644
Male	9 (81%)	93 (83%)	
Female	2 (19%)	14 (17%)	
**Body mass index (kg/m^2), median IIQR)**	-	-	-
**Child-Pugh stage**			-
A	11 (100%)	107 (100%)	
B	0 (0%)	0 (0%)	
**Performance status**			0.382
0	7 (64%)	19 (26%)	
≥1	4 (36%)	55 (74%)	
**Type of immunotherapy**			1.000
PD-(L)1 Monotherapy	11 (100%)	105 (98%)	
Atezolizumab + bevacizumab	0 (0%)	0 (0%)	
Combination therapies	0 (0%)	2 (2%)	

**Immunotherapy as systemic**			0.330
First-line	6 (54%)	39 (36%)	
Second-/third-line	5 (46%)	68 (64%)	
**Macrovascular invasion**	4 (36%)	30 (28%)	0.441
**Extrahepatic metastasis**	6 (54%)	43 (40%)	0.233
**Alpha-fetoprotein (ng/ml)**			0.743
≤200	5 (50%)	60 (57%)	
>200	5 (50%)	44 (43%)	
**AST (U/l) median (IQR)**	-	-	-
**ALT (U/l) median±IQR**	46 (7-943)	31 (11-165)	0.397
Abbreviations: AST, aspartate aminotransferase; ALT, alanine aminotransferase; BCLC, Barcelona-Clinic Liver Cancer; PS, Performance Status; HCC, hepatocellular carcinoma; NAFLD, non-alcoholic fatty liver disease			

Supplementary Table 11: Antibodies for standard mouse flow cytometry

Fluorochrome	Dilution	Name	Clone	Cat #
Alexa Fluor 488	1/400	Donkey anti-rabbit IgG		406416
Alexa Fluor 488	1/200	TCF1/7	C63D9	6444S
Alexa647	1/200	FOXP3	150D	320014
Alexa700	1/200	CD4	RM4-5	100536
APC	1/200	CD11b	M1/70	101212
APC	1/200	CD44	IM7	103012
APC	1/200	IFNy	XMG1.2	505810
APC	1/200	NK1.1	PK136	108710
BV421	1/200	PD-L1	MIH5	564716
BV421	1/200	Blimp-1	5E7	564270
BV421	1/200	Tbet	4B10	644832
BV421	1/200	CD19	6D5	115549
BV421	1/200	CD11b	M1/70	101251
BV421	1/200	CD11c	N418	117343
BV421	1/200	NK1.1	PK136	108741
BV421	1/400	Donkey anti-rabbit IgG		406410
BV510	1/200	CD45	30-F11	103138
BV605	1/200	PD-1	29F.1A12	135220
BV605	1/200	Ly-6G	1A8	127639
BV650	1/200	TCRb	H57-597	742483
BV711	1/200	CD11c	N418	117349
BV711	1/200	CD69	H1.2F3	104537
FITC	1/200	CD19	6D5	115505
FITC	1/200	CD45	I3/2.3	147710
FITC	1/200	IL-10	JES5-16E3	505006
FITC	1/200	Ly-6C	HK 1.4	128005
FITC	1/200	TCR γδ	UC7-13D5	107504
FITC	1/200	NK1.1	PK136	108706
FITC	1/200	IFNy	XMG1.2	505805
FITC	1/200	CD127	A7R34	135007
PE	1/200	CD274	10F.9G2	124307
PE	1/200	CD69	H1.2F3	104508
PE	1/200	F4/80	BM8	123110

PE	1/200	Granzyme B	NGZB	12-8898-80
PE	1/200	TNF	MO6-XT22	506306
PE	1/200	Ly-6C	AL-21	560592
PE	1/200	GZMB	GB11	GRB04
PE	1/50	TOX	REA473	130-120-716
PE/Cy7	1/200	CD3	17A2	100220
PE/Cy7	1/200	NK1.1	PK136	108713
PE/Cy7	1/200	F4/80	BM8	123114
PE/Dazzle	1/200	CD11c	N418	117348
PE/Dazzle	1/200	CD279	RMP1-30	109116
PE/Dazzle	1/200	CD62L	MEL-14	104448
PE/Dazzle	1/200	TNF	MP6-XT22	506346
PE-Cy7	1/200	CD44	IM7	103029
PE-Cy7	1/200	Eomes	Dan11mag	25-4875-82
PE-Cy7	1/200	CD244	2B4	25-2441-82
PerCP/Cy5.5	1/200	CD8a	53-6.7	100734
PerCP/Cy5.5	1/200	I-A/I-E	M5/114.15.2	107625
PerCP/Cy5.5	1/200	LY-6C	HK1.4	128012
	1/300	CD16/32	93	101302
	1/200	Foxo1	C29H4	2880T
eFluor514	1/1000 (2mM)	Calcium Sensor		65-0859-81

Supplementary Table 12: Antibodies for mouse flow cytometry

Marker	Fluorochrome	clone	Company	catalog-nr	Dilution
Zombie aqua	Live dead		Biolegend		1/250
BUV563	CD45	30-F11	BD	565710	1/400
BUV661	CD11B	Mac-1	BD	565080	1/400
BUV805	CD8	53-6.7	BD	612898	1/100
BUV496	CD4	GK1.5	BD	564667	1/100
BV570	CD44	IM7	BioLegend	103037	1/100
PE-Cy5	TCRb	H57-597	Biolegend	109209	1/300
PE-Cy5.5	CD19	ebio 1D3	eBioscience	35-0193-82	1/200
Biotin	NK1.1	PK136	BD	553163	1/100
APC-Cy7	KLRG-1	2F1/KLRG1	BioLegend	138426	1/100
AlexaFlour 647	TIM3	B8.2C12	BioLegend	134005	1/100
PerCP-eFlour710	CD39	24DMS1	eBioscience	46-0391-80	1/200
BV 650	CD25	PC61	BioLegend	102038	1/100
BV 785	PD-1	29F.1A12	BioLegend	135225	1/100
BV 605	CD73	TY/11.8	BioLegend	127215	1/200
BV 421	LAG3	C9B7W	BioLegend	125221	1/100
FITC	GZMB	GB11	BioLegend	515403	1/100
PE-eFlour610	FOXP3	FJK-16s	eBioscience	61-5773-82	1/200
PE-Cy7	Tbet	4B10	BioLegend	644823	1/100
BV480	Ki-67	B56	BD	566109	1/100
BV 711	TNF	MP6-XT22	Biolegend	506349	1/300
PE	Eomes	Dan11mag	eBioscience	12-4875-82	1/200
BUV 737	IFN-g	XMG1.2	BD	564693	1/100
AlexaFlour 700	CTLA4	UC10-4F10-11	BD	565778	1/200
BUV 395	Streptavidin		BD	564176	1/200
Zombie NIR	Live dead		Biolegend		1/250
BUV 563	CD45	30-F11	BD	565710	1/400
BUV 805	CD8a	53-6.7	BD	612898	1/100
BUV 496	CD4	GK1.5	BD	564667	1/200

BV 570	CD44	IM7	BioLegend	103037	1/100
BUV 737	CD62L	MEL-14	BD	565213	1/400
BUV 661	CD11b	M1/70	BD	565080	1/400
BV 785	PD-1	29F.1A12	BioLegend	135225	1/100
PE-Cy5	TCRb	H57-597	Biolegend	109209	1/300
PE-Cy5.5	CD11c	N418	eBioscience	35-0114-82	1/400
BV 421	CD64	X54-5/7.1	BioLegend	139309	1/100
Biotin	CD103	2.00E+07	Biolegend	121404	1/100
FITC	Ly-6C	AL-21	BD	553104	1/800
BV 650	CD206	C068C2	BioLegend	141723	1/100
BV 711	NK1.1	PK136	BioLegend	108745	1/100
APC	PD-L1	10F.9G2	BioLegend	124311	1/150
PE-Cy7	MerTK	DS5MMER	eBioscience	25-5751-82	1/200
AlexaFlour 700	Ly-6G	1A8	BioLegend	127622	1/100
BV 510	F480	BM8	BioLegend	123135	1/100
BB700	MHCII	M5/114.15.2	BD	746197	1/400
PE-eFlour610	FOXP3	FJK-16s	eBioscience	61-5773-82	1/200
BV480	KI67	B56	BD	566109	1/100
BV 605	CD73	TY/11.8	BioLegend	127215	1/200
PE	CD69	H1.2F3	BD	553237	1/100
BUV 395	Streptavidin		BD	564176	1/200

Supplementary Table 13: Antibodies for human flow cytometry

Fluorochrome	Marker	clone	Company	catalog-nr	Diluation
	Fc blocking		Biolegend	422302	1/200
BV510	LD	Zombie aqua	Biolegend		1/250
BUV395	CD45	HI-30	BD	563792	1/200
BUV496	CD19	SJ25C1	BD	612938	1/100
BUV563	CD4	SK3	BD	566000	1/100
BUV661	HLADR	G46-6	BD	565073	1/400
BUV615	CD45RO	UCHL1	BD	Customized	1/200
BUV737	CD39	TU66	BD	564726	1/100
BUV805	CD3	UCHT1	BD	565515	1/100
BV421	PD-1	EH12.2H7	Biolegend	329919	1/50
BV570	CD8	RPA-T8	Biolegend	301038	1/100
BV605	CD127	A019D5	Biolegend	351333	1/40
BV650	CD27	O323	Biolegend	302827	1/100
BV711	CD11b	ICRF44	Biolegend	301343	1/100
PE-Cy5.5	CD45RA	MEM-56	LifeTechnologies	MHCD45RA18	1/150
PE-Cy5	Pangd	IMMU510	Beckman Coulter	IM2662U	1/40
PE/Dazzle™ 594	CD56	5.1H11	Biolegend	362543	1/50
BB630-P2	CD103	BER-ACT8	Biolegend	350220	1/40
PerCP-eFluor® 710	KLRG1	13F12F2	ThermoScientific	46-9488-42	1/100
AF488	TCF1	C63D9	ABCAM	6444S	1/100
BB630-P2	Strep		BD	customized	1/200
BB790-P	CTLA4	BNI3	BD	624296	1/50
A647	TCF1	7F11A10	Biolegend		1/100
A700	GZMB	GB11	BD	560212	1/200
APC-C7	Eomes	WD1928	EBiosciences	47-4877-41	1/50
BV480	Ki67	B56	BD	566109	1/100
BV785	TNF	Mab11	Biolegend	502947	1/300

PE-Cy7	FOXP3	236A/E7	EBiosciences	25-4777-42	1/25
PE	TOX	47	BD	612567	1/100

Fluorochrome	Marker	Clone	Company	catalog-nr	Concentration
Zombie UV	Live dead		Biolegend	423108	1µl/test
APC/Cy7	CD45	HI30	Biolegend	304014	1µl/test
PerCP-Cy5.5	CD3	UCHT1	Biolegend	300430	1µl/test
BV605	CD56	HCD56	Biolegend	318334	3µl/test
AF700	CD16	3G8	Biolegend	302026	3µl/test
BV510	CD15	W6D3	Biolegend	323028	1µl/test
PE/Cy7	CD8	RPA-T8	BD	557746	3µl/test
BV711	PD-1	EH12.2H7	Biolegend	329928	5µl/test

Supplementary Table 14: Antibodies for Immunohistochemistry

Target	Dilution	Clone	Cat #
Ki-67	1/200	RM-9106-S1	RM-9106-S1
CD8	1/200	4SM15	14-0808-82
PD-1	1/100	-	AF1021
PD-L1 (cryo)	1/200	D5V3B	64988
pSTAT1	1/100	58D6	9167
pSTAT3	1/100	D3A7	9145
GP73	1/100	C-15	sc-48011
CD44v6	1/500	9A4	BMS145
GS	1/100	ab16802	Ab16802
Cleaved Caspase 3	1/300	Asp175	9661
Collagen IV	1/30	Cedarlane	CL50451AP-1

Supplementary Table 15: Antibodies used for CyTOF analysis

Channel	Target	Clone	Vendor/ Source	Conjugation Method	Antibody cocktail	Dilution
89Y	CD45	HI30	Fluidigm	n.a.	surface	1:400
104Pd	B2M	2M2	Biolegend	ITCB-EDTA + $^{104}Pd(NO_3)_2$ Trace Sciences	barcode	1:25
105Pd	B2M	2M2	Biolegend	ITCB-EDTA + $^{105}Pd(NO_3)_2$ Trace Sciences	barcode	1:25
106Pd	B2M	2M2	Biolegend	ITCB-EDTA + $^{106}Pd(NO_3)_2$ Trace Sciences	barcode	1:25
108Pd	B2M	2M2	Biolegend	ITCB-EDTA + $^{108}Pd(NO_3)_2$ Trace Sciences	barcode	1:25
110Pd	B2M	2M2	Biolegend	ITCB-EDTA + $^{110}Pd(NO_3)_2$ Trace Sciences	barcode	1:25
111Cd	CD123	6H6	Biolegend	Fluidigm MCP9	surface	1:100
112Cd	CD45RA	4G11	DRFZ	Fluidigm MCP9	surface	1:100
113In	HLA-DR	L243	DRFZ	Fluidigm X8 + $^{113}InCl_3$ (Trace Sciences)	surface	1:100
114Cd	CD4	RPA-T4	Biolegend	Fluidigm MCP9	surface	1:50
115In	CD3	UCHT1	DRFZ	Fluidigm X8 + $^{115}InCl_3$ (Trace Sciences)	surface	1:100
116Cd	CD15	W6D3	Biolegend	Fluidigm MCP9	surface	1:25
139La	CD8	GN11/13 4D7	DRFZ	Abcam/Expedeon 139La Lightning Kit	surface	1:400
140Ce	CD14	RMO52	Beckman Coulter	Fluidigm X8 + $^{140}CeCl_3$ (Trace Sciences)	surface	1:100
141Pr	CD206	15-2	Biolegend	Fluidigm X8	surface	1:100
142Nd	CD19	HIB19	Fluidigm	n.a.	surface	1:100
143Nd	CD117	104D2	Fluidigm	n.a.	surface	1:100
144Nd	CD1a	HI149	Biolegend	Fluidigm X8	surface	1:200
145Nd	CD1c (BDCA1)	AD5-8E7	Miltenyi	Fluidigm X8	surface	1:100
146Nd	TCR Va7.2	3C10	Biolegend	Fluidigm X8	surface	1:50
147Sm	CD279 (PD-1)	EH12.2H7	Biolegend	Fluidigm X8	surface	1:100
148Nd	PD-L1	29E.2A3	Fluidigm	n.a.	surface	1:50
149Sm	CD25	2A3	Fluidigm	n.a.	surface	1:100
150Nd	CD86	IT2.2	Fluidigm	n.a.	surface	1:100
151Eu	CD278/ICOS	C398.4A	Fluidigm	n.a.	surface	1:100
152Sm	CD103	Ber-ACT8	Biolegend	Fluidigm X8	surface	1:200

153Eu	BDCA2	201A	Fluidigm	n.a.	surface	1:50
154Sm	CD163	GHI/61	Fluidigm	n.a.	surface	1:100
155Gd	CD27	2E4	Sanquin	Fluidigm X8	surface	1:800
156Gd	CD183 (CXCR3)	G025H7	Fluidigm	n.a.	surface	1:50
157Gd	CD161	HP-3G10	Biolegend	Fluidigm X8 + $157GdCl_3$ (Trace Sciences)	surface	1:100
158Gd	FceRIa	AER-37 [CRA-1]	Biolegend	Fluidigm X8	surface	1:200
159Tb	CD197 (CCR7)	G043H7	Fluidigm	n.a.	surface	1:50
160Gd	Tbet	4B10	Fluidigm	n.a.	i.c.	1:50
161Dy	NKp46	9E2	Biolegend	Fluidigm X8	surface	1:100
162Dy	FoxP3	PCH101	Fluidigm	n.a.	i.c.	1:50
163Dy	CD294 (CRTH2)	BM16	Fluidigm	n.a.	surface	1:50
164Dy	CD141 (BDCA-3)	AD5-14H12	Miltenyi	Fluidigm X8	surface	1:200
165Ho	CD127 (IL-7Ra)	A019D5	Fluidigm	n.a.	surface	1:100
166Er	CD34	581	Fluidigm	n.a.	surface	1:50
167Er	Gata3	REA174	Miltenyi	Fluidigm X8	i.c.	1:25
168Er	ROR gamma (RORC,NR1F3)	REA278	Miltenyi	Fluidigm X8	i.c.	1:25
169Tm	TCRgd	11F2	Miltenyi	Fluidigm X8	surface	1:50
170Er	CD152 (CTLA-4)	14D3	Fluidigm	n.a.	i.c.	1:100
171Yb	CD68	Y1/82A	Fluidigm	n.a.	i.c.	1:100
172Yb	NKp44	2.29	Miltenyi	Fluidigm X8	surface	1:100
173Yb	Granzyme B	GB11	Fluidigm	n.a.	i.c.	1:400
174Yb	CD94	HP-3D9	Fluidigm	n.a.	surface	1:50
175Lu	Eomes	WD1928	Thermofisher	Fluidigm X8	i.c.	1:200
176Yb	CD56 (NCAM)	NCAM16.2	Fluidigm	n.a.	surface	1:200
194Pt	CD16	3G8	Biolegend	Fluidigm 194Cis-Platinum	surface	1:200
195Pt	CD36	AC106	Miltenyi	Fluidigm 195Cis-Platinum	surface	1:400
196Pt	CD11c	Bu15	Biolegend	Fluidigm 196Cis-Platinum	surface	1:200
198Pt	CD11b	ICRF44	Biolegend	Fluidigm 198Cis-Platinum	surface	1:100
209Bi	CD38	HIT2	Biolegend	Fluidigm X8 + $BiCl_3$ (Sigma-Aldrich)	sf	1:100
