# Supplementary material for: NASH limits anti-tumour surveillance in immunotherapy-treated HCC
Source: Nature. 2021 Mar 24;592(7854):450–6. doi: 10.1038/s41586-021-03362-0 (PMC8046670; doi:10.1038/s41586-021-03362-0)
Supplement: Supplementary file 2 — Reporting Summary [file 41586_2021_3362_MOESM2_ESM.pdf]

nature research

Corresponding author(s): Prof. Dr. Mathias Heikenwälder

Last updated by author(s): Feb 4, 2021

Reporting Summary

Nature Research wishes to improve the reproducibility of the work that we publish. This form provides structure for consistency and transparency in reporting. For further information on Nature Research policies, see Authors & Referees and the Editorial Policy Checklist.

Statistics

For all statistical analyses, confirm that the following items are present in the figure legend, table legend, main text, or Methods section.

n/a	Confirmed	
☐	☒	The exact sample size (*n*) for each experimental group/condition, given as a discrete number and unit of measurement
☐	☒	A statement on whether measurements were taken from distinct samples or whether the same sample was measured repeatedly
☐	☒	The statistical test(s) used AND whether they are one- or two-sided<br>*Only common tests should be described solely by name; describe more complex techniques in the Methods section.*
☐	☒	A description of all covariates tested
☐	☒	A description of any assumptions or corrections, such as tests of normality and adjustment for multiple comparisons
☐	☒	A full description of the statistical parameters including central tendency (e.g. means) or other basic estimates (e.g. regression coefficient) AND variation (e.g. standard deviation) or associated estimates of uncertainty (e.g. confidence intervals)
☐	☒	For null hypothesis testing, the test statistic (e.g. *F*, *t*, *r*) with confidence intervals, effect sizes, degrees of freedom and *P* value noted<br>*Give P values as exact values whenever suitable.*
☒	☐	For Bayesian analysis, information on the choice of priors and Markov chain Monte Carlo settings
☒	☐	For hierarchical and complex designs, identification of the appropriate level for tests and full reporting of outcomes
☐	☒	Estimates of effect sizes (e.g. Cohen's *d*, Pearson's *r*), indicating how they were calculated

Our web collection on statistics for biologists contains articles on many of the points above.

Software and code

Policy information about availability of computer code

Data collection	Data were collected with Microsoft Excel.
Data analysis	Flowcytometry: Cells were analyzed using BD FACSFortessa or BD FACSSymphony and data were analyzed using FlowJo (v10.6.2). For sorting, FACS Aria II and FACSAria FUSION in collaboration with the DKFZ FACS core facility were used. For UMAP/FlowSOM plots, BD FACSymphony data (mouse and human) were exported from FlowJo (v10). Analyses was performed as described elsewhere46.<br>scRNA-seq (mouse): Single-cell capturing for scRNA-seq and library preparation was described previously47. Libraries (pooled at equimolar concentration) were sequenced on an Illumina NextSeq 500 at a median sequencing depth of ~40,000 reads/cell. Sequences were mapped to the mouse (mm10), using HISAT (version 0.1.6); reads with multiple mapping positions were excluded. Reads were associated with genes if they were mapped to an exon, using the Ensembl gene annotation database (Embl release 90). Exons of different genes that shared a genomic position on the same strand were considered as a single gene with a concatenated gene symbol. The level of spurious UMIs in the data was estimated by using statistics on empty MARS-seq wells and excluded rare cases with estimated noise > 5% (median estimated noise overall experiments was 2%). Removal of specific mitochondrial genes, immunoglobulin genes, genes linked with poorly supported transcriptional models (annotated with the prefix “Rp-”), and cells with less than 400UMIs. Gene features were selected using Tvm=0.3 and a minimum total UMI count > 50. Hierarchical clustering of the correlation matrix between those genes (filtering genes with low coverage and computing correlation using a down-sampled UMI matrix) and selected the gene clusters that contained anchor genes. We used K=50, 750 bootstrap iterations, and otherwise standard parameters. Subsets of T-cells were obtained by hierarchical clustering of the confusion matrix and supervised analysis of enriched genes in homogeneous groups of metacells48.<br>scRNA-seq (human): De-multiplexing and barcode processing was performed using the Cell Ranger Software Suite (Version 4.0.0) and reads were aligned to human GRCh3863. Gene-barcode matrix containing cell barcodes and gene expression counts was generated by counting the single-cell 3’ UMIs, imported into R (v4.0.2) where quality control and normalization were executed using Seurat v364. Cells with more than 10% mitochondrial genes, fewer than 200 genes per cell, or more than 6000 genes per cell were excluded. Matrices from 10 samples were integrated with Seurat v3 to remove batch effects across samples. PCA analysis of filtered gene-barcode matrices of all CD3+ cells, visualized by UMAP (top 50 principal components) and identification of major cell types using the highly variable features and indicative markers was performed. Besides, pairwise combinations of CD4+ T-cells vs CD4+PD-1+ T-cells and CD8+ T-cells vs CD8+PD-1+ T-cells were performed using the results of differential expression analysis by DESeq2 (v1.28.1)65, setting CD4+/CD8+ T-cells as controls.

Volcano plots were then generated using EnhancedVolcano (v1.6.0)66 to visualize the results of differential expression analysis.
Velocity and correlation analyses of scRNA-seq data: Velocyto (0.6) was used to estimate the spliced/unspliced counts from the pre-aligned bam files49. RNA velocity, latent time, root, and terminal states were calculated using the dynamical velocity model from scvelo (0.2.2)50. Kendall's rank correlation coefficient was used to correlate the expression patterns of biologically significant genes with latent time.
Mass spectrometry: Analyses was performed using MaxQuant (1.6.7.0), mouse UniProt Isoform fasta (Version: 2019-02-21, number of sequences 25,233) as a source for protein sequences. 1% FDR was used for controlling at the peptide and protein level, with a minimum of two peptides needed for consideration of analysis. Gene set enrichment analysis was performed using ClusterProfiler (3.18)51 and gene sets obtained from WikiPathway (wikipathways.org) and MSigDB (broadinstitute.org/msigdb)52–54.
Isolation of RNA and library preparation for bulk RNA sequencing: RNA isolation23and library preparation for bulk 3'-sequencing of poly(A)-RNA was described previously55. Using the with the Feature Extraction software (11.0.1.1, Agilent Technologies), gencode gene annotations version M18 and the mouse reference genome major release GRCm38 were derived from (https://www.gencodegenes.org/). Dropseq tools v1.1256 were used for mapping the raw sequencing data to the reference genome. Resulting UMI filtered count matrix was imported into R v3.4.4. Prior differential expression analysis with Limma v3.40.657 sample-specific weights were estimated and used as coefficients alongside the experimental groups as a covariate during model fitting with Voom. T-test was used for determining differentially (p-value below 0.05) regulated genes between all possible experimental groups. Gene set enrichment analysis was conducted with the pre-ranked GSEA method53 within the MSigDB Reactome, KEGG, and Hallmark databases (broadinstitute.org/msigdb). Raw sequencing data are available under the accession number PRJEB36747.
Mass cytometry: The sample was acquired on a Helios mass cytometer and raw data were EQ-Bead-normalized using Helios mass cytometer and Helios instrument software (version 6.7). Compensation was performed in CATALYST (v1.86)70 and FlowCore (1.50.0). De-barcoding and gating of single, live CD45+ cells were performed using FlowJo (v10.6.2). Then, data of CD45+ cells were imported into Cytosplore 2.3.1 and transformed using the arcsinh(5) function. Major immune cell lineages were identified at the first level of a 2-level hierarchical stochastic neighbor embedding (HSNE) analysis with default perplexity and iteration settings. HSNE with the same parameters was run on CD3+ cells to identify T-cell phenotypes. Gaussian mean shift clustering was performed in Cytosplore and a heatmap of arcsinh(5)-transformed expression values of all antibody targets was generated. Cell type identification was based on the transformed expression values and clusters showing high similarity were merged manually.
Search strategy, selection criteria, and meta-analysis of phase III clinical trials: The literature search was done through MEDLINE on PubMed, Cochrane Library, Web of Science, and clinicaltrials.gov, using the following searches: "checkpoint inhibitors", "HCC", "phase III", between January 2010 and January 2020, and complemented by hand searches of conference abstracts/presentations. Single-center, non-controlled trials, studies with insufficient data to extract hazard ratios (HR), 95% confidence intervals, or trials including disease entities other than HCC were excluded. As conference abstracts were not excluded, quality assessment of the included studies was not performed. Three studies5,13,14 fulfilled the criteria and were included in the quantitative synthesis (Extended Data 10). The primary outcome of the meta-analysis was OS, defined as the time from randomization to death. HRs and CIs related to OS were extracted from the papers/conference presentations5,13,14. Pooled HRs were calculated using the random-effects model (Der Simonian and Laird), and the generic inverse variance was used for calculating weights72. To evaluate heterogeneity among studies, Cochran's Q test and I2 index were used. A p-value < 0.10 in the Q-test was considered to indicate substantial heterogeneity. I2 was interpreted as suggested in the literature: 0% to 40% might not represent significant heterogeneity; 30% to 60% may represent moderate heterogeneity, 50% to 90% may represent substantial heterogeneity, 75% to 100% represents considerable heterogeneity. All statistical pooled analyses were performed using the RevMan 5.3 software.
Statistical analyses: Data was collected in Microsoft Excel. Mouse data are presented as the mean±SEM. Pilot experiments and previously published results were used to estimate the sample size, such that appropriate statistical tests could yield significant results. Statistical analysis was performed using GraphPad Prism software version 7.03 (GraphPad Software). Exact p-values lower than p< 0.1 are reported and specific tests are indicated in the legends. Survival analyses were performed using IBM SPSS Statistics version 25 (SPSS Inc., Chicago, IL).

For manuscripts utilizing custom algorithms or software that are central to the research but not yet described in published literature, software must be made available to editors/reviewers. We strongly encourage code deposition in a community repository (e.g. GitHub). See the Nature Research guidelines for submitting code & software for further information.

Data

Policy information about availability of data

All manuscripts must include a data availability statement. This statement should provide the following information, where applicable:
- Accession codes, unique identifiers, or web links for publicly available datasets
- A list of figures that have associated raw data
- A description of any restrictions on data availability

The proteomics data described in this article is available at ProteomeXchange Consortium via the PRIDE under the identifier XD017236 login/password: /37SciglLe. The bulk-RNA-seq data described in this article is available at PRJEB36747. The single-cell RNA-seq data described in this article is available at GEO Submission (GSE144635). The array of comparative genomic hybridization data described in this article is available at GEO Submission (GSE144875). The results here are in whole or part based upon data generated by the TCGA Research Network: https://www.cancer.gov/tcga. The human single-cell RNA-seq data described in this article is available at GEO Submission (GSE159977). Databases used in this manuscript are WikiPathways (wikipathways.org), MSigDB (www.broadinstitute.org/msigdb).

Field-specific reporting

Please select the one below that is the best fit for your research. If you are not sure, read the appropriate sections before making your selection.

☒ Life sciences ☐ Behavioural & social sciences ☐ Ecological, evolutionary & environmental sciences

For a reference copy of the document with all sections, see nature.com/documents/nr-reporting-summary-flat.pdf

Life sciences study design

All studies must disclose on these points even when the disclosure is negative.

Sample size	Pilot experiments and previous published results were used to estimate the sample size such that appropriate statistical tests could yield significant results (DOI: 10.1016/j.ccell.2014.09.003; DOI: 10.1038/s41591-019-0379-5). The exact n numbers used in the study are indicated in the source data file.
Data exclusions	No data exlusion of mice and human.
Replication	All experiments presented were conducted with sufficient mouse numbers to ensure statistical significance could be reached, particularly for experiments involving tumor studies.<br>Biochemical or image based data were reproduced in multiple mice: e.g. Weight analysis of mice, Measuring transaminase levels, NASH phenotype characterization, HCC development characterization, flow cytometry analyses, immunohistochemical staining, confocal analysis.<br>All attempts of replicating data were successfull.
Randomization	5-week-old C57Bl/6 mice were randomly allocated into different groups and were fed with appropriated diet or treatment regimens.<br>No prospective trial was performed, therefore randomization of human specimen was not relevant for the performed experiments.
Blinding	Experiments with different genotypes on the same diet were blinded.<br>For the dissection between ND or NASH mouse experiments this was not possible since the type of diet and the systemic obesity were visible to the researcher, e.g., the color of the normal diet is brown and color of the NASH-diet is green.<br>The analysis of the transaminase level was blinded and measured with code labeling. The NASH phenotype analysis was blinded - still the morphology of the normal/chow-fed liver is different from a NASH liver.

Reporting for specific materials, systems and methods

We require information from authors about some types of materials, experimental systems and methods used in many studies. Here, indicate whether each material, system or method listed is relevant to your study. If you are not sure if a list item applies to your research, read the appropriate section before selecting a response.

Materials & experimental systems

n/a	Involved in the study	
☐	☒	Antibodies
☒	☐	Eukaryotic cell lines
☒	☐	Palaeontology
☐	☒	Animals and other organisms
☐	☒	Human research participants
☐	☒	Clinical data

Methods

n/a	Involved in the study	
☒	☐	ChIP-seq
☐	☒	Flow cytometry
☒	☐	MRI-based neuroimaging

Antibodies

Antibodies used	Description of all antibodies (clone and supplier) used in the study are provided in the Materials&Methods Table 11-15.
Validation	Validation of commercial antibodies was done on a regular quality control of each lot by the manufacturer (e.g. Biolegend "The antibody was purified by affinity chromatography and conjugated with PE under optimal conditions."; "Each lot of this antibody is quality control tested by immunofluorescent staining with flow cytometric analysis."; "Every lot of product is quality tested against a "gold standard" reference lot. A new lot is only released based on our defined QC specifications to ensure lot to lot reproducibility and reliability. BioLegend guarantees the stability and performance of all our products shipped at room temperature". or Bioxcell for in vivo treatment antibodies "Binding Validation: Western Blot data shown below confirms that htis clone binds to its target antigen").

Animals and other organisms

Policy information about studies involving animals; ARRIVE guidelines recommended for reporting animal research

Laboratory animals	All animals are described in the Supplementary Materials.<br>All mice were male and on a C57Bl/6 background and were put from 5-8 weeks of age onwards in the respective diets and/or treatment regimens.<br>Following strains were used for the experiment described in the manuscript: C57Bl6/J and Pdcd1-/-.
Wild animals	not used

Field-collected samples	not used
Ethics oversight	Regierungspräsidium Karlsruhe, Karlsruhe, Germany

Note that full information on the approval of the study protocol must also be provided in the manuscript.

Human research participants

Policy information about studies involving human research participants

Population characteristics	Human characteristics are reported in Supplementary Materials Table 1-10.
Recruitment	No active recruitment was done in the frame of this study Baseline characteristics of analyzed human specimen are listed in Supplementary Tables 1-10.<br>The human survival analyses are retrospective studies, they need prospective validation.
Ethics oversight	The respective ethics committee is listed in the Material&Method section.<br>Flow cytometry of patient material (Figure 3a,b) was performed under ethics oversight: BIOFACS Study KEK 2019-00114.<br>Flow cytometry of human samples (Extended Data 9d) was approved by the local ethical committee (AC-2014-2094 n 03).<br>High-throughput RNA sequencing of human specimen: As previously reported, RNA sequencing analysis was performed using the data from 206 snap-frozen biopsy samples from 206 patients diagnosed with NAFLD in France, Germany, Italy, and the UK and enrolled in the European NAFLD Registry (GSE135251)58,59. Collection and use of data of the European NAFLD Registry were approved by the relevant local and/or national Ethical Review Committee58.<br>Isolation of cells for single-cell RNA-seq data analysis (human): Analyses of liver samples from patients undergoing bariatric surgery at the Department of Surgery at Heidelberg University Hospital (S-629/2013).<br>Histological and immunohistochemical analysis of NASH/HCC cohort: All samples were obtained from International Genomic HCC Consortium with IRB approval.<br>A cohort of patients with HCC treated with PD-(L)1-targeted immunotherapy: The retrospective analysis was approved by local Ethics Committees. Data from this cohort were published previously73.<br>Validation cohort: Ethical approval to conduct this study was granted by the Imperial College Tissue Bank (Reference Number R16008).<br>Analysis of human samples from the Department of Pathology and Molecular Pathology, University Hospital Zurich, was approved by the local ethics committee (‘Kantonale Ethikkommission Zürich’, KEK-ZH-Nr. 2013-0382 and BASEC-Nr. PB_2018-00252).

Note that full information on the approval of the study protocol must also be provided in the manuscript.

Clinical data

Policy information about clinical studies

All manuscripts should comply with the ICMJE guidelines for publication of clinical research and a completed CONSORT checklist must be included with all submissions.

Clinical trial registration	This study was performed not in active clinical trials.
Study protocol	This study was performed not in active clinical trials.
Data collection	The respective data collection (fresh/retrospective cohort analysis/analyses of published data) process is listed in the Material&Method section.
Outcomes	This study was performed not in active clinical trials.

Flow Cytometry

Plots

Confirm that:

☒ The axis labels state the marker and fluorochrome used (e.g. CD4-FITC).

☒ The axis scales are clearly visible. Include numbers along axes only for bottom left plot of group (a 'group' is an analysis of identical markers).

☒ All plots are contour plots with outliers or pseudocolor plots.

☒ A numerical value for number of cells or percentage (with statistics) is provided.

Methodology

Sample preparation	Please see Supplementary Materials.<br>Mice were transcardially perfused with PBS, and livers were dissected. Livers were incubated for up to 35min in 37°C with digestion buffer (Collagen IV 1:10 (60 U f.c.) and DNase I 1:100 (25µg/ml f.c.)) and subsequently passed through a 100µm filter. Livers were washed with RPMI1640 (#11875093) medium and subsequently centrifuged for 7min/300g/4°C. Lymphocyte enrichment was achieved by a 2-step Percoll gradient (20ml 25% Percoll/HBSS underlay with 20ml 50% Percoll/HBSS) and centrifugation for 15min/1800g/4°C (Acc:1 Dcc:0). Leukocytes were collected, washed with HBSS, centrifuged for 10min/700g/4°

C, counted and transferred to a 15ml Falcon for a final washing step with FACS buffer (PBS supplemented with v/v 0.4% 0.5M EDTA pH= 8 and w/v 0.5% albumin fraction V (#90604-29-8)). Isolation of splenic lymphocytes was done by passing spleens through a 100µm mesh and subsequent washing. Afterwards, an erythrocyte lysis using ACK-buffer 1x 2ml for 5 min RT and then a wash was performed. For T-cell re-stimulation, cells were incubated for 2h, 37°C, 5% CO2 in RPMI 1640 supplemented with v/v 2% fetal calf serum using 1:500 Biolegend´s Cell Activation Cocktail (with Brefeldin A) (#423304) and 1:1000 Monensin Solution (1,000X) (#420701). Staining was performed using Live/Dead discrimination by using DAPI or ZombieDyeNIR according to the manufacturer´s instructions. After washing (~400g, 5min, 4°C), cells were stained in 25µl of titrated antibody master mix for 20min at 4°C and washed again (antibodies shown in Table 7-9). Samples for flow cytometric activated cell sorting (FACS) were then sorted. Samples for flow cytometry were fixed using eBioscience IC fixation (#00-8222-49) or Foxp3 Fix/Perm kit (#00-5523-00) according to the manufacturer´s instruction. Intracellular staining was performed in eBioscience Perm buffer (#00-8333-56). Cells were analyzed using BD FACSFortessa or BD FACSSymphony and data were analyzed using FlowJo. For sorting, a FACS Aria II and a FACSAria FUSION in collaboration with the DKFZ FACS core facility were used.
Analysis of human material (Table 1) was performed on human liver tissue samples (needle biopsies or resected tissue, BIOFACS Study KEK 2019-00114), that were processed within 4 hours after collection. Tissue samples were minced using scalpels, incubated (1 mg/mL collagenase IV (Sigma Aldrich), 0.25 µg/mL DNase (Sigma Aldrich), 10% FCS (Thermo Fisher Scientific), RPMI 1640 (Seraglob)) for 30 min at 37oC with continuous shaking. The enzymatic reaction was stopped with 2 mM EDTA (StemCell Technologies, Inc) in PBS. The homogenized cell suspension was filtered through 100 µm cell strainer and centrifuged to get a cell pellet. Next cells were resuspended in FACS Buffer (PBS, EDTA 2mM, FCS 0.5%) with Human TruStain FcX™ (Fc Receptor Blocking Solution) (Biolegend) and incubated for 15 min at 4oC. After cells were spun without washing and stained with the flow cytometry antibodies mix and Zombie Aqua™ Fixable Viability Kit (Biolegend).

Instrument

Cells were analyzed using BD FACSFortessa or BD FACSSymphony. For sorting, a FACS Aria II and a FACSAria FUSION in collaboration with the DKFZ FACS core facility were used.

Software

Collected data was analyzed by FlowJo V10.2.

Cell population abundance

Absolute quantification by using CountBright™ Absolute Counting Beads.

Gating strategy

Debry esclusion byFSC-A/SSC-A. Doublets were excluded by using FSC-A/
FSC-H and SSC-A/SSC-H gates. Life/Dead exclusion was performed. Remaining cells were analyzed according to displayed markers.

☒ Tick this box to confirm that a figure exemplifying the gating strategy is provided in the Supplementary Information.
