# Supplementary material for: NASH limits anti-tumour surveillance in immunotherapy-treated HCC
Source: Nature. 2021 Mar 24;592(7854):450–6. doi: 10.1038/s41586-021-03362-0 (PMC8046670; doi:10.1038/s41586-021-03362-0)
Supplement: Supplementary file 3 — Gating strategy flow cytometry. [file 41586_2021_3362_MOESM3_ESM.pdf]

Myeloid Gating strategy

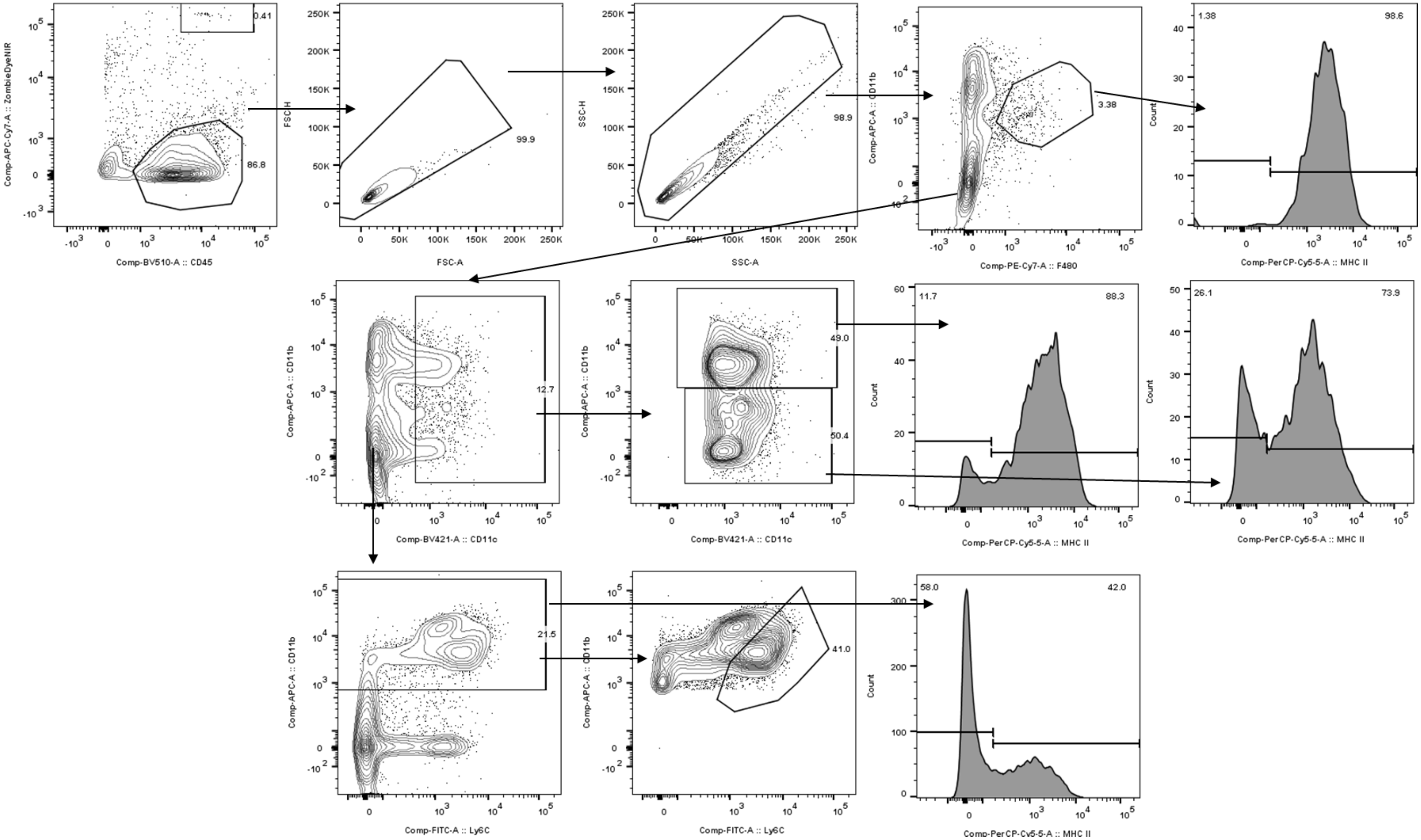

Myeloid Gating strategy

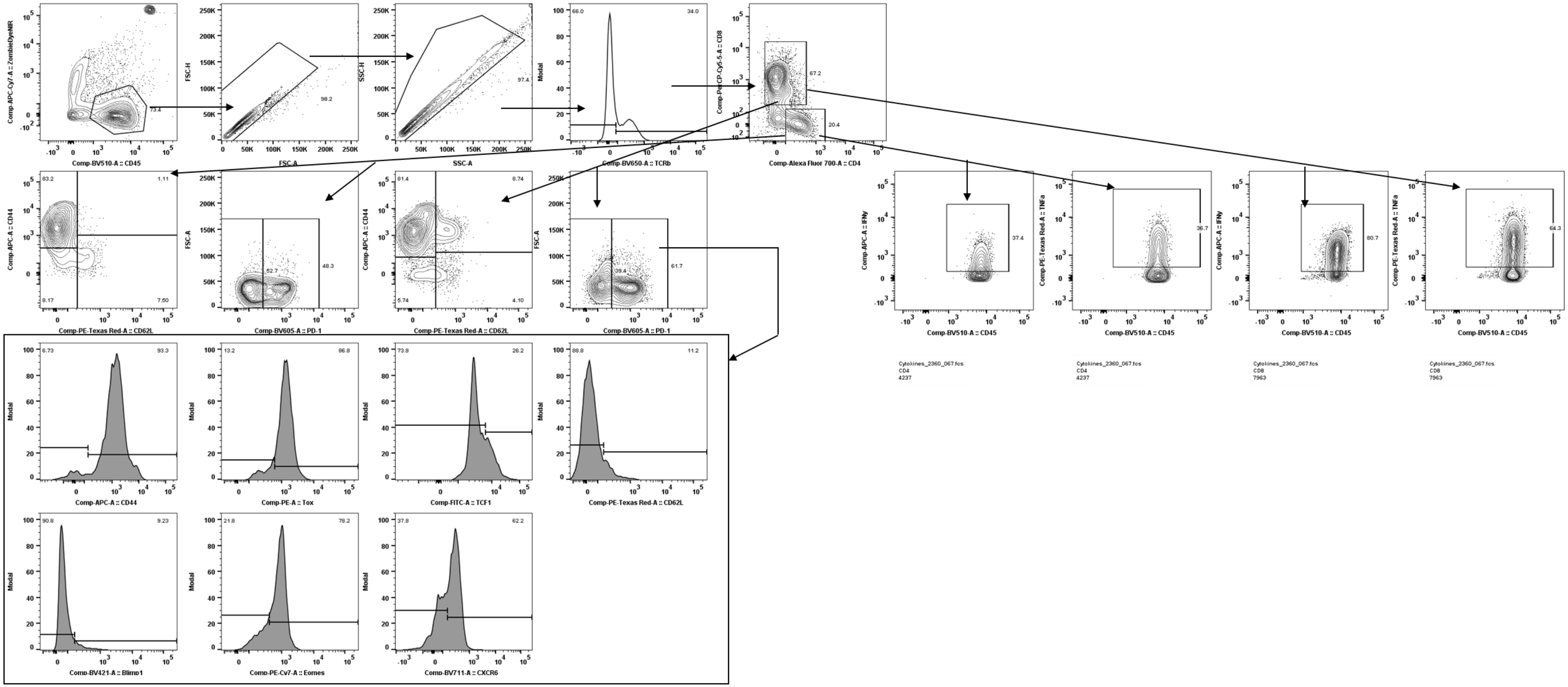

Published gating strategies

- Gaiting strategy can be also found in Malehmir et al., 2019; https://www.nature.com/articles/s41591-019-0379-5
- Extended Data 9a-c (human gaiting strategy) can be found in https://journals.lww.com/ctg/Fulltext/2019/07000/Immunologic_Features_of_Patients_With_Advanced.3.aspx
